# Supplementary material for: Subtle changes in chromatin loop contact propensity are associated with differential gene regulation and expression
Source: Nat Commun. 2019 Mar 5;10:1054. doi: 10.1038/s41467-019-08940-5 (PMC6401380; doi:10.1038/s41467-019-08940-5)
Supplement: Supplementary file 1 — Supplementary Information [file 41467_2019_8940_MOESM1_ESM.pdf]

Subtle changes in chromatin loop contact propensity are associated with differential gene regulation and expression

Greenwald and Li et. al.

Supplementary Information

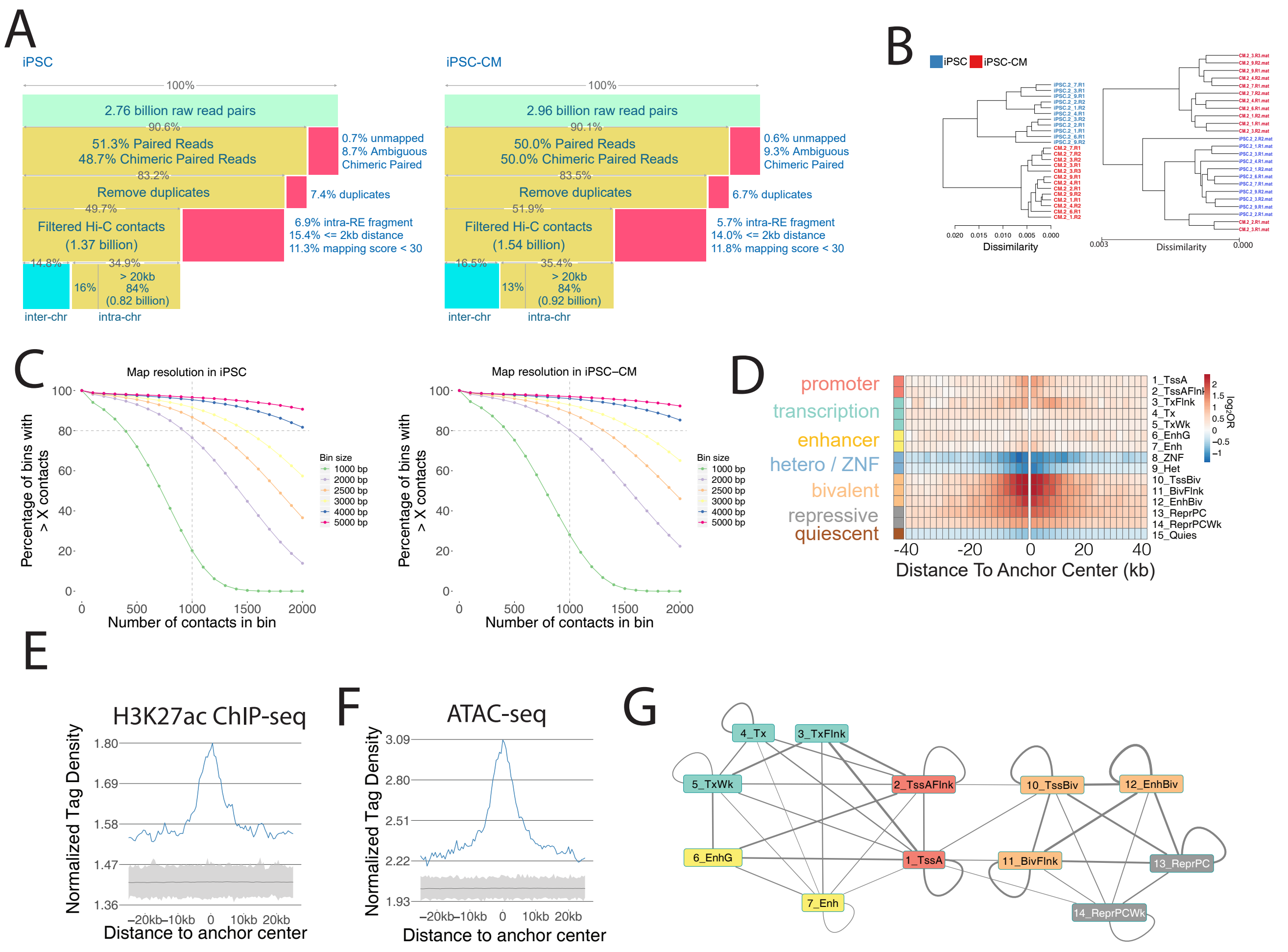

Supplementary Figure 1: (A) Summary statistics for Hi-C data processing using Juicer pipeline. Green boxes indicate the overall raw read pairs obtained from the Hi-C experiments. Yellow boxes indicate read pairs or contacts retained after each filtering step. Red boxes indicate read pairs removed by filtering. Blue boxes indicate the final retained inter-chromosomal contacts. The percentage values between the boxes represent percentage of read pairs calculated based on the total number of raw read pairs, and the percentage values within the rectangles represent percentages relative to the total number in the box. (B) Hierarchically clustered dendrogram of Hi-C contact matrix distances at 1 MB resolution with Pearson Correlation (left) and HiC Rep (right). iPSC lines shown blue, iPSC-CM lines shown in red, lactate purified iPSC-CM sample is shown in pink. (C) Map resolutions at different bin sizes, defined as the resolution at which 80% of loci have 1000 or more contacts with any other locus, indicated by color in iPSCs (left) and iPSC-CMs (right). (D) Enrichment of 15 ROADMAP chromatin states around the center of reference loop anchors in iPSC (using the E020 iPSC tissue from Roadmap) in an 80kb window with 2kb intervals. (E-F) Average normalized tag densities from H3K27ac ChIP-seq (E) and ATAC-seq (F) around loop anchors in iPSC. (G) Network diagram showing two discrete subnetworks of iPSC chromatin states at iPSC called loops, with edges connecting statistically significant pairs of chromatin states found at opposing anchors. The thickness of the edge indicates the odds ratio of significance, and the presence or absence of an edge indicates statistical significance.

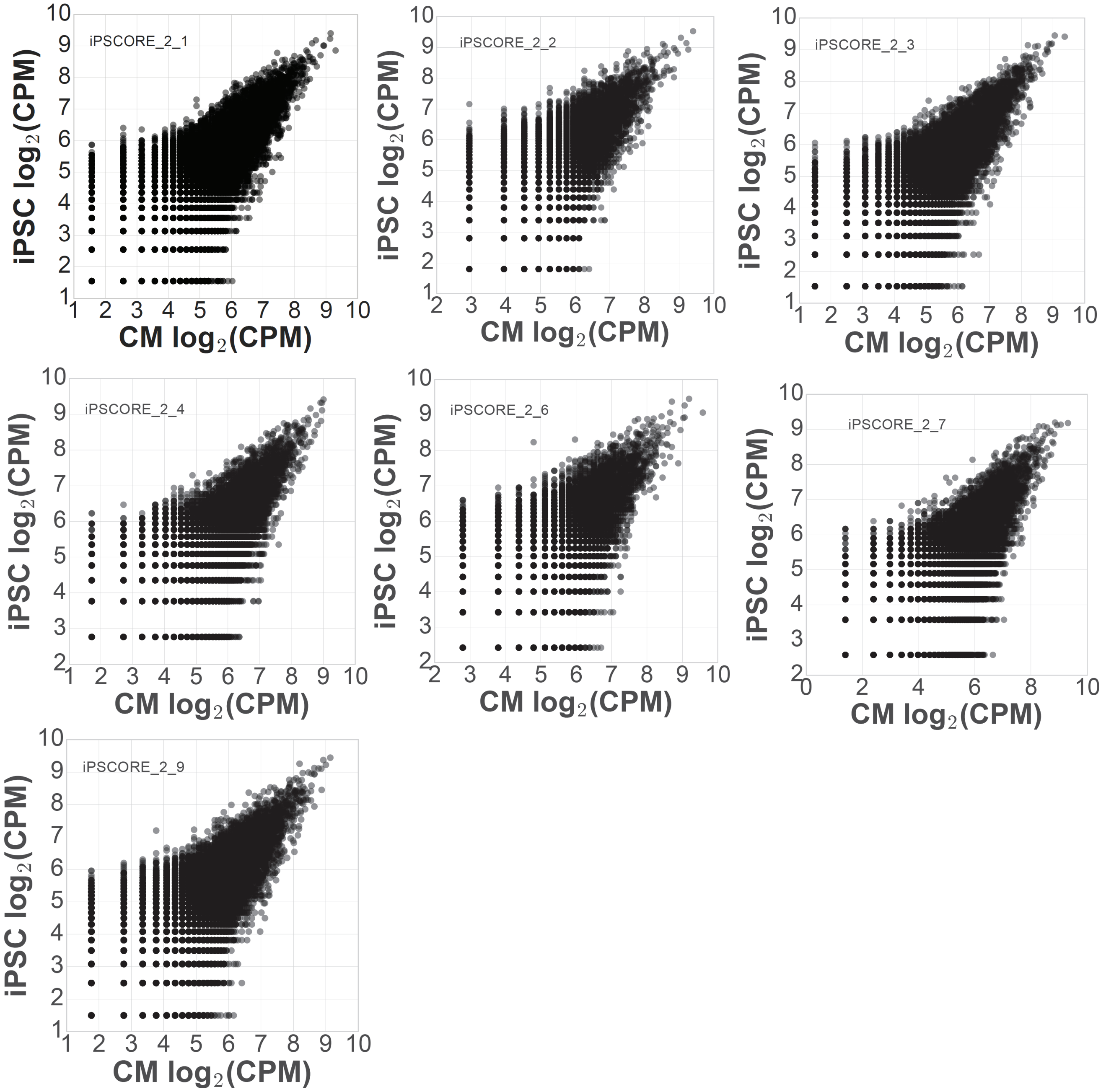

Supplementary Figure 2: Individual logCPM plots for iPSC vs iPSC-CM for each individual. A consistent pattern of loops with high logCPMs in both cell types is observed; at low logCPMs, a banding pattern is observed, consistent with less data and thus less resolution in the count data.

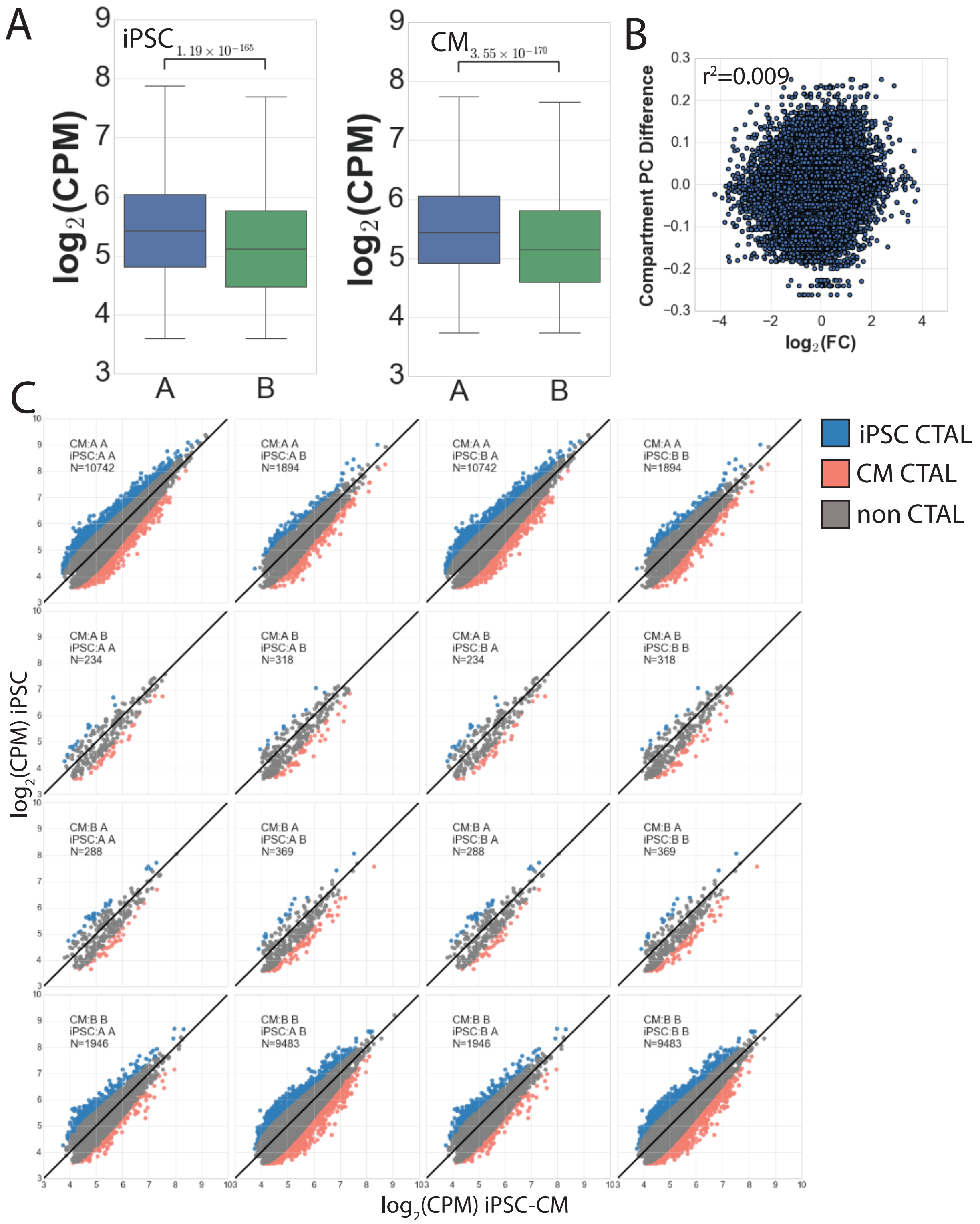

Supplementary Figure 3: (A) Boxplot (all four quartiles shown via lower whisker, lower half of box, upper half of box, and upper whisker; lines indicate median; outliers not shown) showing the difference in logCPMs across A and B compartments for iPSC (left) and iPSC-CM (right) called loops. (B) Scatter plot of $\log_2$(Fold Change) vs compartment PC difference across cell types. (C) logCPM plots for iPSC vs iPSC-CM for each possible combination of compartments at each anchor. Similar patterns in plot shapes indicate that compartment differences are not driving CTALs.

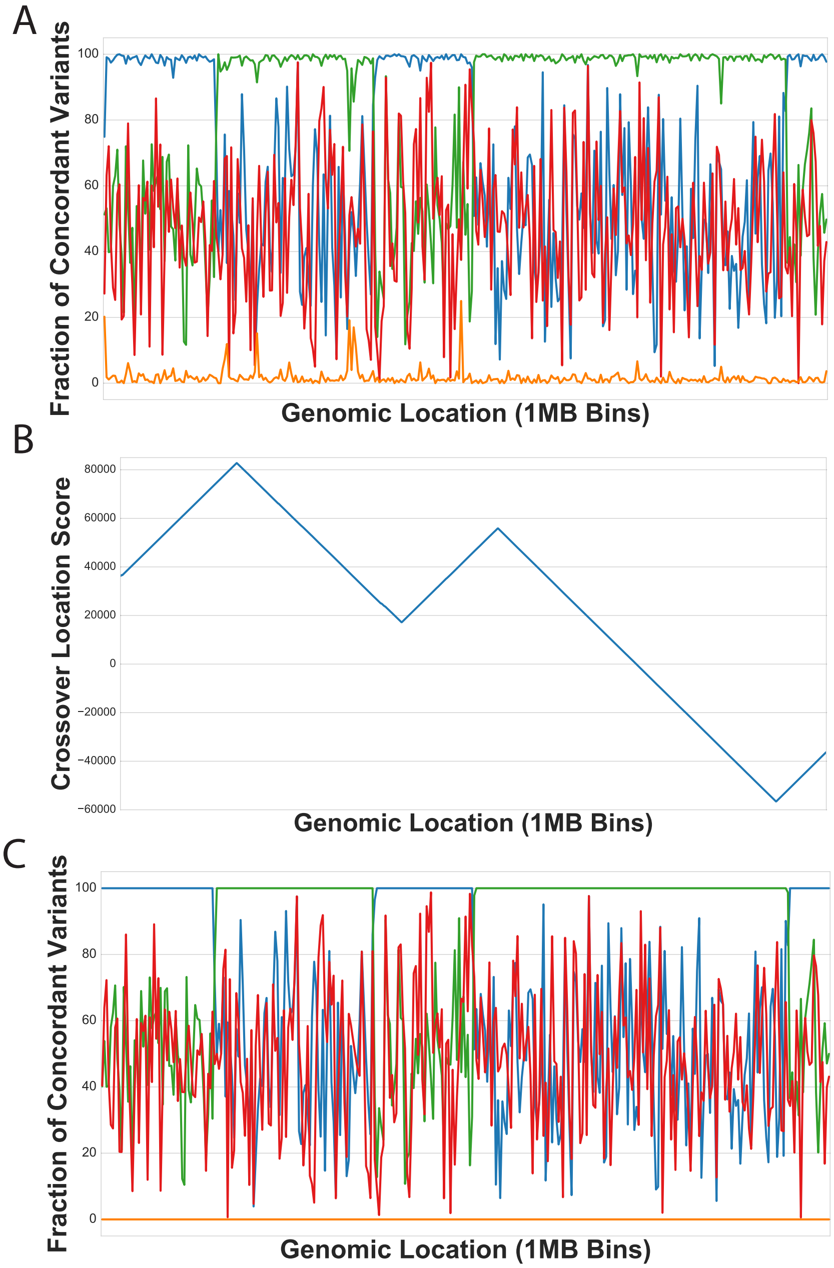

Supplementary Figure 4: (A) Fraction of concordant heterozygous variants for each parental haplotype (maternal haplotype 1: red; maternal haplotype 2: orange; paternal haplotype 1: blue; paternal haplotype 2: green) before leveraging family information on chromosome 6, in 1MB bins across the chromosome. The haplotype color hovering around 100% indicates one child haplotype, and the haplotype color hovering around 0% indicates the other child haplotype, as if a heterozygous variant matches the maternal haplotype, the other allele at the same SNV must not match the maternal haplotype, by definition. The noise around 100% and 0% indicates the baseline point error rate from Haploseq, and the switches from blue to green indicates a crossover even that occurred within the father. Note that the two haplotypes from the parents that were not inherited by the child (red and blue/green) have a mean ~50%, as expected with variants matching by random chance. (B) Crossover location score calculated per SNV across the genome, plotted in as the average in 1MB bins. Extreme points indicate crossover events at the blue to green transitions in panel A. (C) Concordance rates after identifying and fixing switch errors, and removing genotyping error SNVs from the family. The random matching of the non-inherited haplotypes remains constant with a mean ~50%.

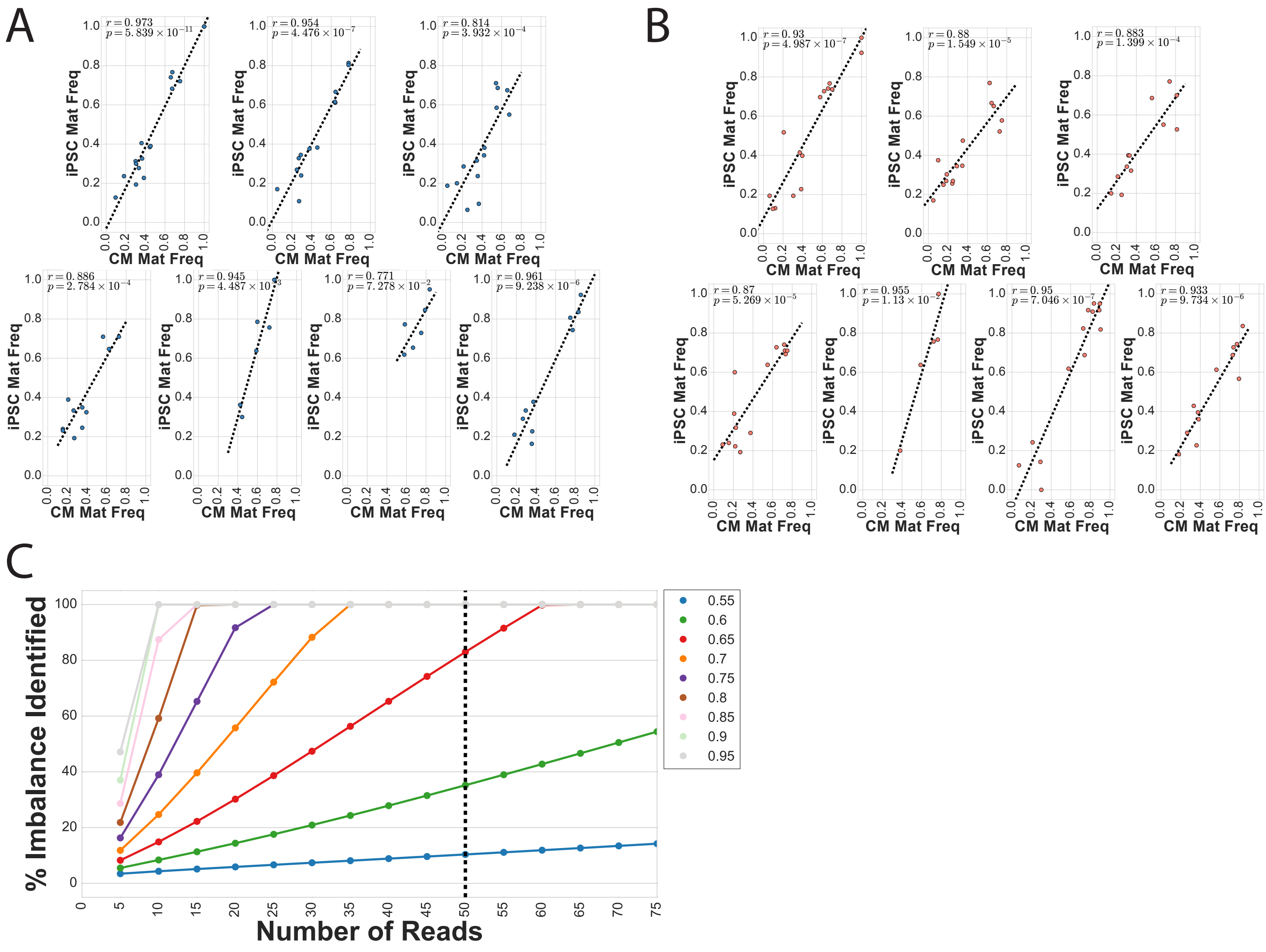

Supplementary Figure 5: (A-B) Scatter plots showing comparison between iPSC and iPSC-CM maternal haplotype frequencies for each of the seven individuals at HTALs identified in either (A) iPSCs or (B) iPSC-CMs. Linear regression correlation and p-value are reported for each cell type for each individual. (C) Percent of allelic imbalance we are powered to detect within one individual at $p<0.05$ at different allelic imbalance fractions (shown in legend on plot). The median loop with imbalance with an effect size of 70% or higher we are powered to detect.

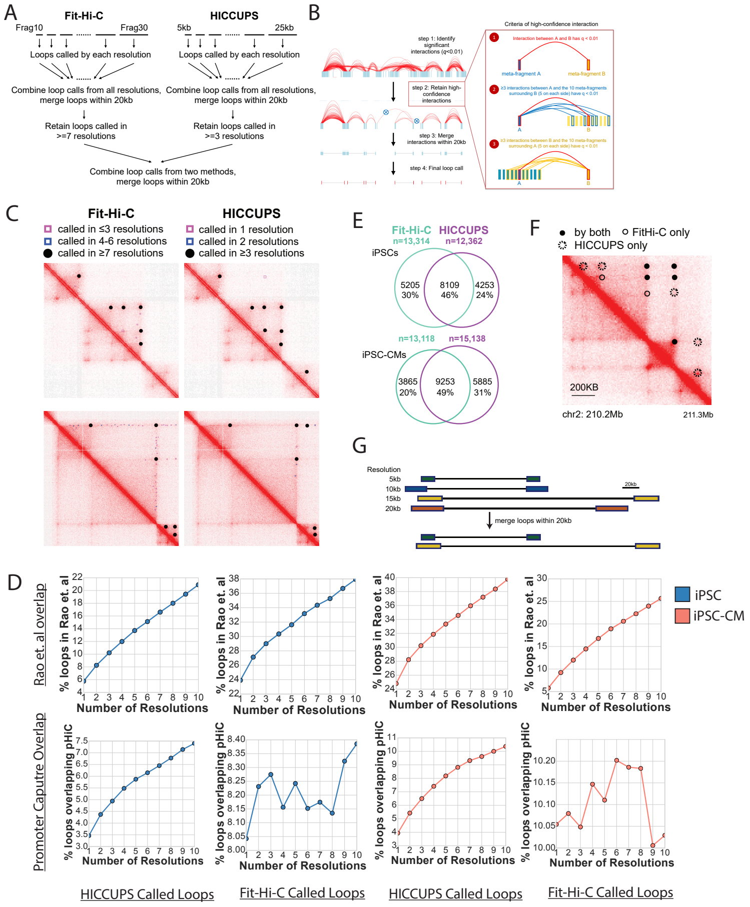

Supplementary Figure 6: (A) Flowchart of the overall loop calling procedures. Chromatin loops in iPSC and iPSC-CM were called using both Fit-Hi-C and HICCUPS. For Fit-Hi-C, loops were called in meta-fragment resolutions that each contained a fixed number of consecutive RE fragments, ranging from 10 to 30 RE fragments (Frag10 to Frag30) followed by combining loops from different resolutions and filtering. For HICCUPS, loops were called using fixed-size bin resolutions from 5kb to 25kb at 1kb bin size intervals followed by combining loops from different resolutions and filtering. (B) Loop calling procedures of Fit-Hi-C. Step 1 shows all the significant (q<0.01) Fit-Hi-C interaction calls between two meta-fragments as red curves, and gray bars underneath them indicate the regions involved in chromatin interactions. Step 2 shows interactions retained after applying the high-confidence interaction criteria. A high-confidence interaction example between meta-fragments A (dark blue bar) and B (dark yellow bar) is shown in the right panel (each curved line indicates a significant interaction with q<0.01; red line indicates the interaction between A and B; blue lines indicate significant interactions between A and meta-fragments surrounding B [light yellow bars]; and yellow lines indicate significant interactions between B and meta-fragments surrounding A [light blue bars]). The interactions supported by only one high-confidence interaction are removed (circled crosses). Step 3 shows interacting loci via merging nearby interactions within 20kb. Step 4 shows the final loop call by selecting the most significant high-confidence interaction within each merged loop set. (C) Hi-C heatmaps with loops shown for each caller at different filtering criteria. These images are high resolution; zooming on the PDF is encouraged. (D) Venn Diagram showing overlap of Fit-Hi-C and HICCUPS loop calls for the iPSC called loops (top) and iPSC-CM called loops (bottom). (E) HiC heatmap showing example loops uniquely called by each caller, and loops called by both callers. (F) To merge across resolutions and calling methods, we intersected the loops sets, retaining the loop with the smallest total anchor size at each intersection event.

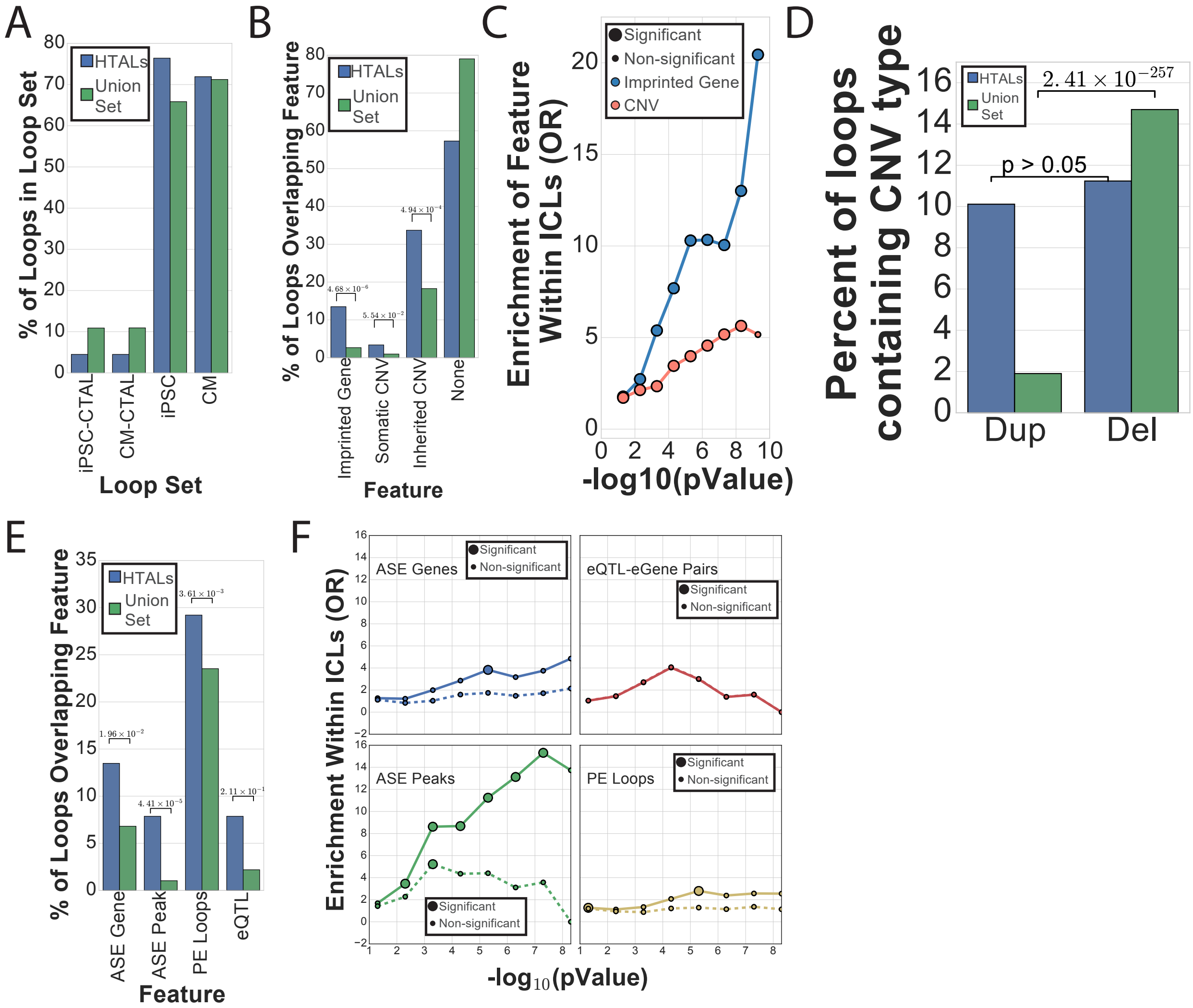

Supplementary Figure 7: A replication of Figure 6 with the p-values and HTALs called using the beta-binomial test from WASP. All results held consistent. (A) Barplot showing the percent of union loops (green) or HTALs (blue) contained within each loop-set. (B) Barplot showing the percent of union loops (green) or HTALs (blue) containing the given genomic feature within it (i.e. the genomic feature overlapped the region between the start of the first anchor and the end of the second anchor). P-values were found via a Fisher's exact test. (C) Line plot showing odds ratio from a Fisher's exact test for HTAL enrichment above the union set for containing an imprinted gene (blue) or containing either an inherited or somatic CNV (red) as a function of the $-\log_{10}$ of the HTAL imbalance p-value. Large circles indicate that the test was significant after Bonferroni correction, and small circles indicate a non-significant association. (D) Barplot showing the percentage of union loops (green) or HTALs (blue) containing only deletions or only duplications. P-values were calculated using a binomial approximation to a normal distribution, adjusted for the number of identified CNVs which were deletions vs duplications. (F) Barplot showing the percent of union loops (green) or HTALs (blue) overlapping the given genomic feature at an anchor. P-values were found via a Fisher's exact test. (G) Line plot showing odds ratio from a Fisher's exact test for HTAL enrichment above the union set for containing the labelled feature as a function of the $-\log_{10}$ of the HTAL imbalance p-value, for either all loops (solid lines), or loops that do not contain an imprinted gene or CNV (dashed lines). Large circles indicate that the test was significant after Bonferroni correction, and small circles indicate a non-significant association.

Supplementary Table 1. Information about individuals used in this study

Subject ID	Subject UUID	Sex	Age	Ethnicity
iPSCORE_2_1	f772212a-cd98-40f9-bdcd-e75740c1f6be	Female	18	Asian / European
iPSCORE_2_2	83dacb11-4180-4807-b099-05fd1561a722	Female	21	Asian / European
iPSCORE_2_3	e932e556-59a6-4f70-9b4c-ef5f69dac3ce	Female	48	Asian / European
iPSCORE_2_4	bd04a8cc-5d63-45bc-a2cc-91b0c7cb6e01	Female	47	Asian / European
iPSCORE_2_6	ae3637d4-493e-482e-bc48-6915e30ffb9c	Female	74	European
iPSCORE_2_7	6a784dd7-9c48-4841-93f9-930b9de49cc4	Male	77	Asian
iPSCORE_2_9	f549b5fa-a6c0-49fb-8a07-dda4f72ff076	Male	52	European

Supplementary Table 2. Hi-C summary statistics

Hi-C sample ID	Total read pairs	Filtered contacts	Inter-chromasomal	Intra-chromosomal	
				ShortRange (<20Kb)	LongRange (>20Kb)
iPSC.2_1.R1	340772001	137882604	30.8%	10.9%	58.2%
iPSC.2_1.R2	210302080	107833819	24.9%	11.0%	64.1%
iPSC.2_2.R1	237313666	112728648	30.6%	10.7%	58.7%
iPSC.2_2.R2	208950060	106581287	24.6%	10.5%	64.9%
iPSC.2_3.R1	199576249	110213471	18.4%	15.7%	65.9%
iPSC.2_3.R2	268989031	145985725	40.2%	9.6%	50.2%
iPSC.2_4.R1	213568883	117885743	28.3%	9.1%	62.7%
iPSC.2_6.R1	291875421	124295183	21.0%	12.8%	66.3%
iPSC.2_7.R1	223504105	109070838	20.0%	12.2%	67.9%
iPSC.2_9.R1	233845655	124497724	18.6%	14.6%	66.8%
iPSC.2_9.R2	333376697	175834534	53.9%	6.5%	39.6%
CM.2_1.R1	277766346	130686176	26.4%	10.4%	63.2%
CM.2_1.R2	226762604	120678692	35.5%	8.1%	56.4%
CM.2_2.R1	242284539	137244976	39.2%	7.9%	52.8%
CM.2_3.R1	207776780	111623429	34.2%	8.8%	57.0%
CM.2_3.R2	214736506	105979008	32.6%	9.5%	57.8%
CM.2_3.R3	166068314	87930498	38.7%	6.8%	54.6%
CM.2_4.R1	249218697	134862865	31.3%	8.2%	60.5%
CM.2_4.R2	272801550	130335621	38.8%	8.2%	53.0%
CM.2_6.R1	210054269	118523936	30.5%	8.5%	61.0%
CM.2_7.R1	223241087	119512780	22.8%	10.3%	66.9%
CM.2_7.R2	256158307	118423157	24.6%	10.9%	64.5%
CM.2_9.R1	198942415	108927983	29.6%	8.3%	62.2%
CM.2_9.R2	214198919	112918260	29.2%	9.6%	61.2%
**Total**	**5722084181**	**2910456957**	**Average: 30% (0.88B)**	**10% (0.29B)**	**60% (1.74B)**

Supplementary Table 3. Loops containing putatively novel imprinted regions

Loop Index
174
2241
2709
2738
2744
7285
8279
9131
15120
15824
16776
19083
19086
19090
19353

Supplementary Table 4. Parameters used for HICCUPS loop calling

**Bin size**	**Peak size (p)**	**Window size (i)**	**Loop merging distance**
5000	4	7	20000
6000	4	6	20000
7000	3	6	20000
8000	3	5	20000
9000	3	5	20000
10000	2	5	20000
11000	2	5	22000
12000	2	5	24000
13000	2	5	26000
14000	2	5	28000
15000	2	5	30000
16000	2	5	32000
17000	2	5	34000
18000	2	5	36000
19000	2	5	38000
20000	2	5	40000
21000	2	5	42000
22000	2	5	44000
23000	2	5	46000
24000	2	5	48000
25000	2	5	50000
