# Supplementary material for: Subtle changes in chromatin loop contact propensity are associated with differential gene regulation and expression
Source: Nat Commun. 2019 Mar 5;10:1054. doi: 10.1038/s41467-019-08940-5 (PMC6401380; doi:10.1038/s41467-019-08940-5)
Supplement: Supplementary file 3 — Description of Additional Supplementary Files [file 41467_2019_8940_MOESM3_ESM.pdf]

Description of Additional Supplementary Files

- Supplementary Data 1: iPSC called-chromatin loops
- Supplementary Data 2: iPSC-CM Called Chromatin Loops
- Supplementary Data 3: Union loops and CTAL status
- Supplementary Data 4: Phased HiC contact counts
- Supplementary Data 5: iPSC-CM Expression Imbalance p-values
- Supplementary Data 6: iPSC-CM Expression Maternal Allele Ratios
- Supplementary Data 7: iPSC Expression Imbalance p-values
- Supplementary Data 8: iPSC Expression Maternal Allele Ratios
- Supplementary Data 9: Map of Loop anchors to Gene Promoters
- Supplementary Data 10: iPSC-CM H3K27AC Allelic Imbalance p-values
- Supplementary Data 11: iPSC-CM H3K27AC Maternal Allele Ratios
- Supplementary Data 12: iPSC H3K27AC Allelic Imbalance p-values
- Supplementary Data 13: iPSC H3K27AC Maternal Allele Ratios
- Supplementary Data 14: Map of Chromatin Loop Anchors to H3K27AC peak
- Supplementary Data 15: Information about samples used in this study
